# Supplementary material for: Digital twin simulation modelling shows that mass testing and local lockdowns effectively controlled COVID-19 in Denmark
Source: Commun Med (Lond). 2024 Oct 4;4:192. doi: 10.1038/s43856-024-00621-9 (PMC11452704; doi:10.1038/s43856-024-00621-9)
Supplement: Supplementary file 2 — Supplementary Information [file 43856_2024_621_MOESM2_ESM.pdf]

Supplementary Information to: Digital twin simulation modelling shows that mass testing and local lockdowns effectively controlled COVID-19 in Denmark

Kaare Græsbøll[1,2*], Rasmus Skytte Eriksen[1], Carsten Kirkeby[3] and Lasse Engbo Christiansen[1,2]

[1*]Department of Epidemiology Research, Statens Serum Institut, Artillerivej 5, Copenhagen S, 2300, Denmark.
[2]Dynamical Systems, Compute, Technical University of Denmark, Anker Engelunds Vej 101, Kongens Lyngby, 2800, Denmark.
[3]Department of Veterinary and Animal Sciences, Faculty of Health and Medical Sciences, University of Copenhagen, Grønnegårdsvej 8, Frederiksberg C, Copenhagen, DK-1870, Denmark.

*Corresponding author(s). E-mail(s): kgrb@ssi.dk;
Contributing authors: rske@ssi.dk; ckir@sund.ku.dk; lsec@ssi.dk;

Appendix A Supplementary Methods

Here we present detailed information of the individual based model (popIBM) used to model the spread of SARS-CoV-2 in Denmark in 2021, including parameter estimates.

A.1 Vaccination

The rollout of SARS-CoV-2 vaccines in Denmark started on December 28th, 2020 with an initial focus on the elderly, on persons with increased risk of serious COVID-19 disease, and on healthcare personnel. The relative scarcity of vaccines in the early period after the vaccines received “Conditional marketing authorization” by the European Medicines Agency (EMA) resulted in a staggered mass-vaccination strategy: The Danish populace was initially divided

Supplementary Table A1 Vaccination target groups in Denmark as of July 1st 2021

Target group	Description
1	Residents in nursing homes etc.
2	Citizens aged over 65 who receive both practical assistance and personal care
3	Citizens aged over 85
4	Selected frontline staff in the healthcare sector, the elderly care sector, and parts of the social services sector
5	Selected patients at particularly high risk, e.g. cancer patients, severely immunosuppressed patients and people with very severe cardiopulmonary disease or liver disease
6	Selected relatives of people at particularly high risk
7	Citizens aged 80-84 years
8	Citizens aged 75-79 years
9	Citizens aged 65-74 years
10A	Citizens aged 60-64 years
10B	Citizens aged 55-59 years
10C	Citizens aged 50-54 years
10D1	Citizens aged 16-19 years and 45-49 years
10D2	Citizens aged 20-24 years and 40-44 years
10D3	Citizens aged 25-30 years and 35-39 years
10D4	Citizens aged 30-34 years
11	Citizens aged 12-15 years

into 12 prioritized groups (later incrementally increased to 18) based on factors such as age and medical history, but also other factors such as whether they worked in the healthcare sector or in other critical positions (the latter group was eventually dropped from consideration)[1]. This grouping of the populace gave granular control over the vaccination rollout where the Danish population was progressively offered a vaccination program as their group began vaccination. The vaccination targets group in Denmark as of July 1st, 2021 is specified in Supplementary Table A1. The temporal distribution of the first vaccination dose is shown in Supplementary Figure A1.

Initially, only the Comirnaty vaccine (BNT162b2) from Pfizer-BioNTech was part of the Danish mass-vaccination strategy, but, once approved by EMA, the Spikevax vaccine (mRNA-1273) from Moderna and to some extent Vaxzevria (AZD1222) from AstraZeneca saw prolific use in the Danish vaccination effort. Towards the later stages of the initial mass-vaccination rollout, the Jcovden vaccine (Ad26.COV2.S) from Jannsen also saw some use in the Danish vaccination landscape[2].

On March 11th, 2021, the Vaxzevria vaccine from AstraZeneca was pulled from the program due to reports of blood clots in vaccinated individuals[2,

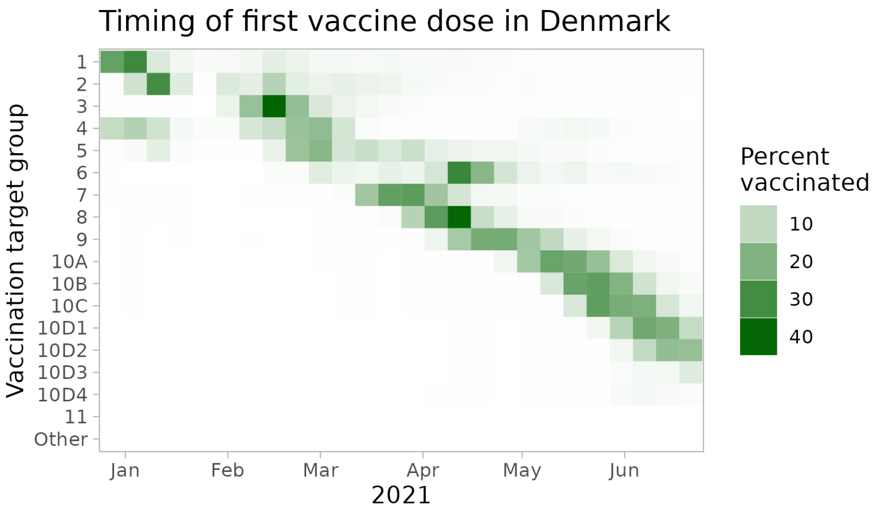

Supplementary Figure A1 Timing of the first vaccine doses in Denmark until July 1st, 2021. The figure shows the percentage of first vaccine doses within each vaccination target group over the first half of 2021.

3]. Up until this point, vaccination with Vaxzevria was primarily offered to healthcare personnel. Those who had their vaccination program interrupted by the hold on Vaxzevria were later offered a mRNA vaccine (Comirnaty or Spikevax) as their second vaccination dose and later additional doses.

The vaccines were made available free of charge to all persons living in Denmark and with vaccination centers distributed nationwide. Denmark achieved a high vaccination coverage (26 percent of the population are fully vaccinated by July 2021), with a vaccine composition that is 80.8 percent 2-dose Comirnaty, 7.7 percent 2-dose Spikevax, 8.8 percent cross-vaccination of Vaxzevria and 1 dose Comirnaty or Spikevax and 2.4 percent 1-dose Jcovden.

Vaccine parameters are discussed in the model parameter section A.5.

A.2 Activity matrices

The activity matrices in the model, $\mathbf{A}(t)$, are based on activity matrices describing a fully open society multiplied element-wise by a time-varying degree of openness. The following provides further details of the framework.

First, activity matrices for an open society were created. As activity data were not readily available from Denmark, we used British data that we assume is similar to Danish conditions. A study by the British Broadcasting Corporation[4], hereafter referred to as the “BBC Pandemic study”, quantified the number of contacts from each age group to each other on a random day. The BBC Pandemic study reports contact matrices for workplaces, schools, homes, and other places. Within each of these groupings, the contacts are further divided into conversational and physical contacts. Inspired by Klepac et al.[5] it was decided to combine the conversational and physical contacts with

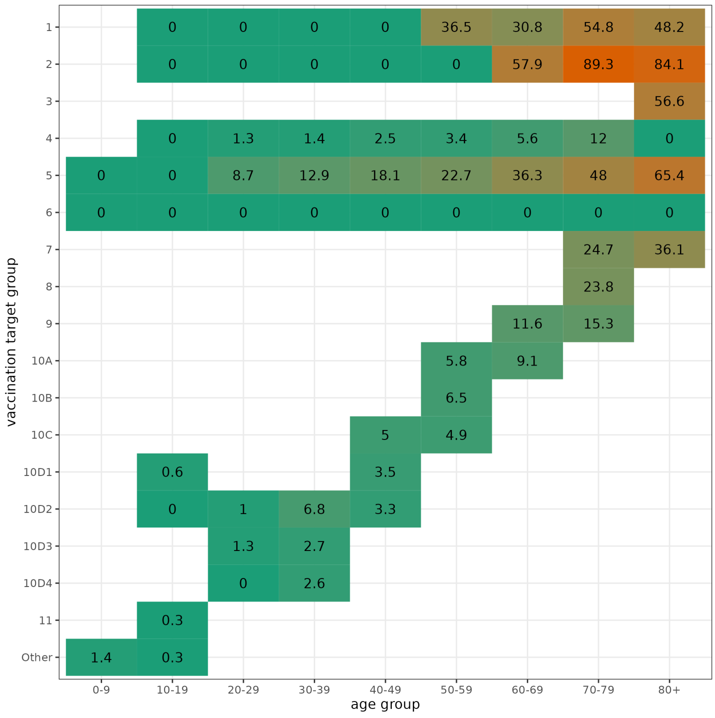

Supplementary Figure A2 Probability, in percent, of going to hospital following an infection in unvaccinated individuals. Probability is reduced by 75% for vaccinated individuals. In some vaccination target groups (e.g. no. 5) risk of hospitalization may be < 0.05 resulting in a displayed 0, this can be driven by the group having very few members, and such the risk is not very well established. However, as there are few members this will also affect the simulations very little, as this is reflected in the demography of the model.

weights 0.2 and 1, respectively to form the activity matrices. Contacts among children below the age of 12 were not included in the BBC study and thus it was necessary to impute these estimates. We decided to use data from the POLYMOD project[6] for these missing age groups. The POLYMOD data are based on a similar study from 2008 that contains fewer study participants. To impute the activity matrices, we assume that children from the age of 5 to 19 have similar contact rates to children of their own age across the two studies (i.e. the diagonal elements of the contact matrices). Therefore, we decided to rescale the POLYMOD matrices to have the same mean along the diagonal as those of the 12-19 year age groups in the BBC matrices and impute the missing elements of the activity matrices with these rescaled values. Using the method described by the BBC Pandemic study [4] the contact matrices were transformed into the 16 five-year age groups up to and including +75-year-old. This results in 16x16 contact matrices for each of the four types of contacts seen in Supplementary Figure A3.

Second, the level of openness for each of the four categories of contacts and age groups was calculated for each change of restrictions in society. Each

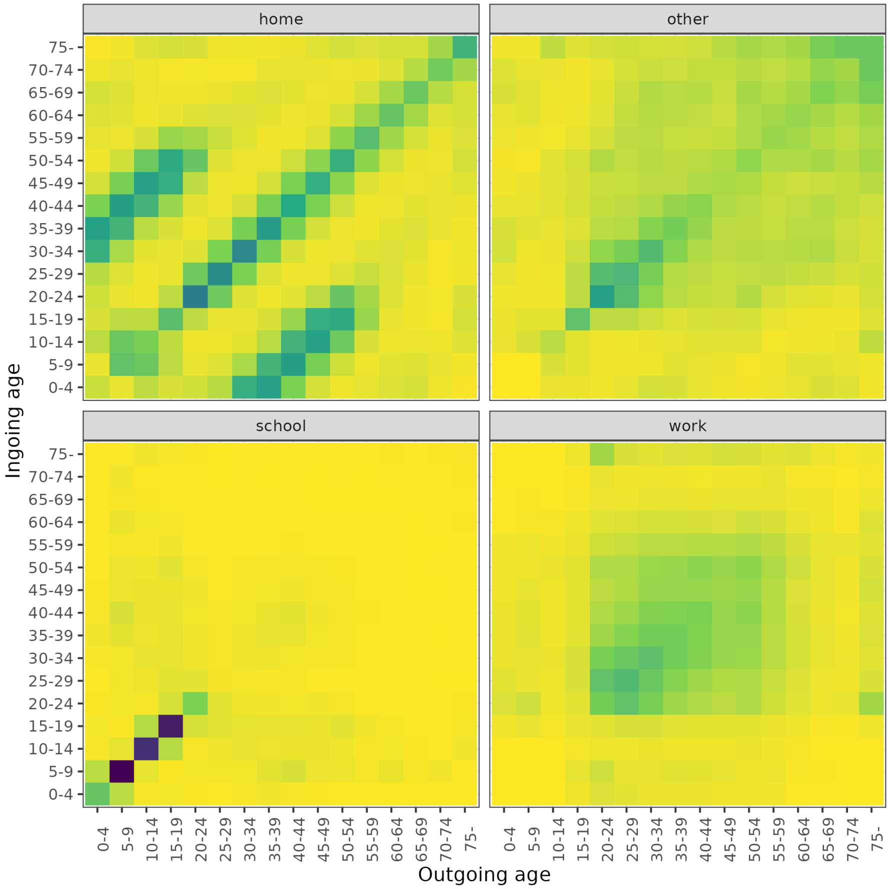

Supplementary Figure A3 Contact matrices for each of the four types of contacts in an open society.

change in restrictions was decomposed into a set of "activity units", representing the activity of e.g. 4th and 5th grade school classes. This way, changes in restrictions are modeled by adding (opening) or subtracting (closing) activity units relative to the previous level of restrictions. Here, the level of restrictions covers both formal restrictions in society as the closing of schools and shops as well as recommendations to the public, e.g. working from home and social distancing. Furthermore, changes in activity unrelated to non-pharmaceutical interventions (e.g. holidays) are in the model handled similarly through the addition and subtraction of activity units.

Each activity unit consists of a vector for each of the four types of contacts where the elements of these vectors describe the proportion of the activity in an open society that is covered by this activity for each of the 16 age groups. Furthermore, each activity unit contains a relative risk for the given activity

unit but does not reflect the proportion of individuals partaking in the activity. A large proportion of the private workforce was not directly affected by restrictions. However, guidelines were given to reduce the risk of transmission at workplaces by e.g. social distancing, disinfection, etc. These changing guidelines were included by adding an additional physical relative risk factor at workplaces.

A full list of restrictions in Denmark is published by Munch et al. [7].

A.3 Municipalities and Parishes

Denmark consists of 98 municipalities which hold approximately 2,100 church parishes. The number of parishes changes slowly over time and is also subject to different definitions. In the model, there are 99 municipalities including the "municipality" of Christiansø, which is not technically a municipality due to being under military command, even though it holds a civilian population. The model includes 2,141 parishes.

Municipalities range in size from 100 to 628,800 persons (rounded to the nearest hundred as population numbers fluctuate in time), median size is 42,800. Parishes range in size from <100 to 46,500 persons (rounded to the nearest hundred as population numbers fluctuate in time), median size is 1,000.

The α parameter was fitted in March 2021, by observing how the shape of the curve describing hospitalizations changed.

A.3.1 Parish risk factor as function of parish cumulative incidence

The risk factor for each parish, ρ_p, was initially set as the observed cumulative incidence of the individual parishes from the beginning of the epidemic to April 26th, 2021 and later normalized (see below). Supplementary Figure A4 shows a histogram of the cumulative incidence of parishes. The figure only covers 90% of Danish parishes as the specific number of test positives was not available to the authors at the time of the parameter estimation due to General Data Protection Regulation (GDPR) restrictions. However, the missing parishes are typically very small and only contain 1.4% of the Danish population. Risk factors for parishes with missing incidences were set to the median risk factor of the parishes that were assessed. Parishes with no infections in this time period were given the lowest risk factor among the other parishes.

When all risk factors were distributed among individuals in the model, they were normalized by dividing by the sum of the risk factors divided by the number of individuals, $\rho_i = N\rho_i / \left(\sum_{i=1}^{N} \rho_i\right)$. This was done after the model had already been calibrated, and doing this normalization ensured that the infection pressure would be on average the same as prior. Following this the risk factor was transformed by cubic root and then normalized again, $\rho_i = N\rho_i^{1/3} / \left(\sum_{i=1}^{N} \rho_i^{1/3}\right)$. The cubic transformation was done to match the observed variation in incidence at the parish level with the simulated.

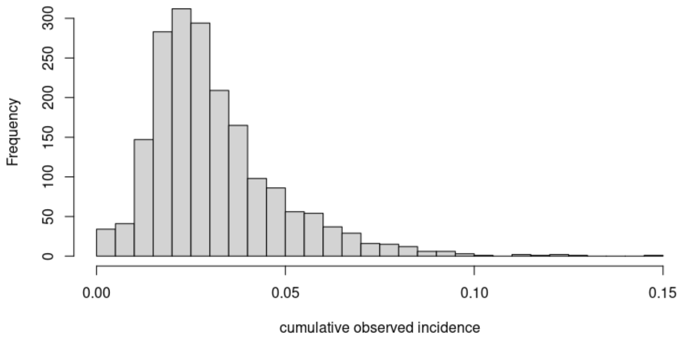

Supplementary Figure A4 Histogram of the fraction of population in a parish that has tested positive for SARS-CoV-2.

A.3.2 Test intensity affected by municipality incidence

From data across the study period, it was observed that test frequency changed according to municipality incidence[8]. Retrospectively, this correlation can be observed to change across the study period. So that prior to local lockdowns (January - February) an increase of 10% in the number of tests could be observed for an increase of 260 positive per 100,000 inhabitants in the municipality. But during the period with local lockdowns (March - July), this would change to a 10% increase in the number of tests for each increase of 120 positive per 100,000 inhabitants. When the model parameters were set in April of 2021, this function was set to a 10% increase in the number of tests for each increase of 130 positives per 100,000 inhabitants. When implementing this in the model total number of tests would be identical to the actual number of tests performed in each group of age and vaccination status, but the geographical distribution of tests would be scaled depending on incidence.

A.4 PCR and antigen tests

The number of tests performed varied over time. Supplementary Figure A5 the fraction of each group tested per day in the period is plotted in the 'mass test' scenarios, for the 'limited tests' scenarios y-axis can be multiplied with 10%.

A.5 Model parameters

As mentioned in the discussion many parameters were not precisely established at the time of the selection, therefore values selected in Table A2 do not match exactly to the values in the references given but reflect the values used in the model.

It can be noted that the used vaccine efficiency (VE) is a rounded number in the vicinity of the number reported in the reference. This is due to the

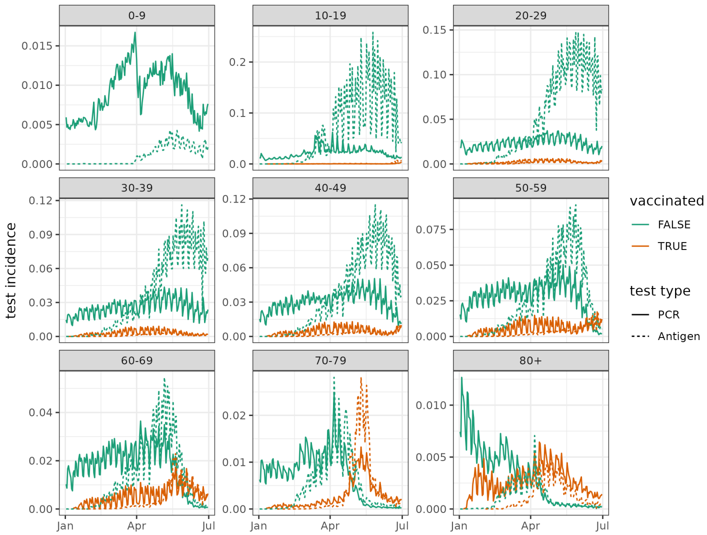

Supplementary Figure A5 Fraction of each age group tested on any given day in the study period. The colors green and orange respectively denote whether the tested individual has been vaccinated or not. The line types, solid and dashed, respectively denote whether the test was a PCR or antigen test.

Supplementary Table A2 Model parameters

Parameter	Symbol	Value	Reference
Weight of municipality transmission	α	.90	A.3
transmission risk	β_0	0.7 1/day	fitted
Seasonal behavior	$\beta(T)$	function	A.6.2
Asymptomatic cases	-	50%	[9]
Average time of being exposed	$1/\gamma_E$	2.5 days	[10]
Average time of being infectious	$1/\gamma_I$	5.3 days	[11]
Average time to symptoms	$1/\gamma_{\text{symp}}$	2 days	[10]
Openness of geographical entity x	$\Delta^x(\text{inc}_x)$	function	A.6.1
Relative activity under lockdown	$\delta(t)$	function	A.6.1
Activity in individual following positive test	ξ	0	assumption
Risk factor for parishes	ρ_p	factor	A.3.1
Activity in society	$\mathbf{A}(t)$	function	A.2
Comirnaty and Spikevax VE against Alpha variant	V	90%	[12, 13]
Vaxzevria VE against Alpha variant	V	60%	[14]
All vaccines, VE against hospitalization	-	75%	[15]

VE = Vaccine efficiency. inc = seven-day incidence per 100,000 inhabitants.

VE used was based on the knowledge available at the time of the making of the model (spring 2021). The VE was often based on preprint results and/or estimates from the technical reports provided by Statens Serum Institute and the UK Health Security Agency [15, 16]. Furthermore, it was also often the case that VE in the population was a little lower than reported in the clinical studies.

All vaccination types reduced the risk of going to hospital by 75% relative to a base level of risk based on observed risk (as described in the Method section) stratified by age and vaccination target group (see Figure A2). Vaccine effects were estimated based at available resources at the time, see Chapter 5 in the technical report from March 2021 [15].

For $1/\gamma_E + 1/\gamma_{\text{symp}} = 4.5$ days which is close to the 4.96 days reported by Galmiche et al. [10].

In this model an infectious period, $1/\gamma_I$, of 5.3 days is used. Kissler et al. report that Ct values vary greatly over the infectious period and that Ct values are below 40 for 9.6 days [11]. However, cutoff Ct values for infectious periods have been in many instances set to lower values. Likely also most of the transmission occurs around the peak period. For this reason, a sensitivity study was performed to check the effect of this period on the results of the study (Section A.7).

A.6 Adaptive behavior

Adaptive behavior may be any change that results in changes in the transmission probability of COVID-19. In the popIBM two forms of adaptive behavior are present. One direct in the form of changing behavior when municipalities or parishes are close to lockdown limits (described in A.6.1) and one indirect from changing behavior with regards to the changes in season (described in A.6.2).

A.6.1 Change in activity close to lockdown limits

During the study period exceeding official incidence limits would prompt local authorities to implement local lockdowns [17]. Trying to avoid lockdowns municipalities and parishes would introduce softer measures when getting close to the lockdown limits. This has been implemented in the model by a piece-wise linear function that increases prior to the incidence limits in the geographic entity reaching the lockdown limits. The incidences and incidence limits were publicly available during the study period. The incidence limits changed over time. Both effects are visualized in Figure A6. Lockdowns would be lifted if the are had been consistently under the limit for seven days, except when incidence limits changed, then they could be lifted immediately [17].

The Figure A6 shows when lockdowns are in full effect (lockdown scale = 1). Effectively this means that $\Delta^x(\text{inc}_x) = 1 - (1 - \delta(t)) \cdot \text{lockdown scale}(\text{inc}_x)$.

A.6.2 Seasonal variation in transmission rate

Seasonal variation in transmission dynamics may be driven by many different factors both behavioral (e.g. social activities moving from inside to outside, or amount of ventilation in private rooms) or related to virus survivability/transmissibility (e.g. humidity, UV radiation). In this work, temperature is taken as a proxy for all these effects, and the seasonal behavior is modeled as an effect of temperature.

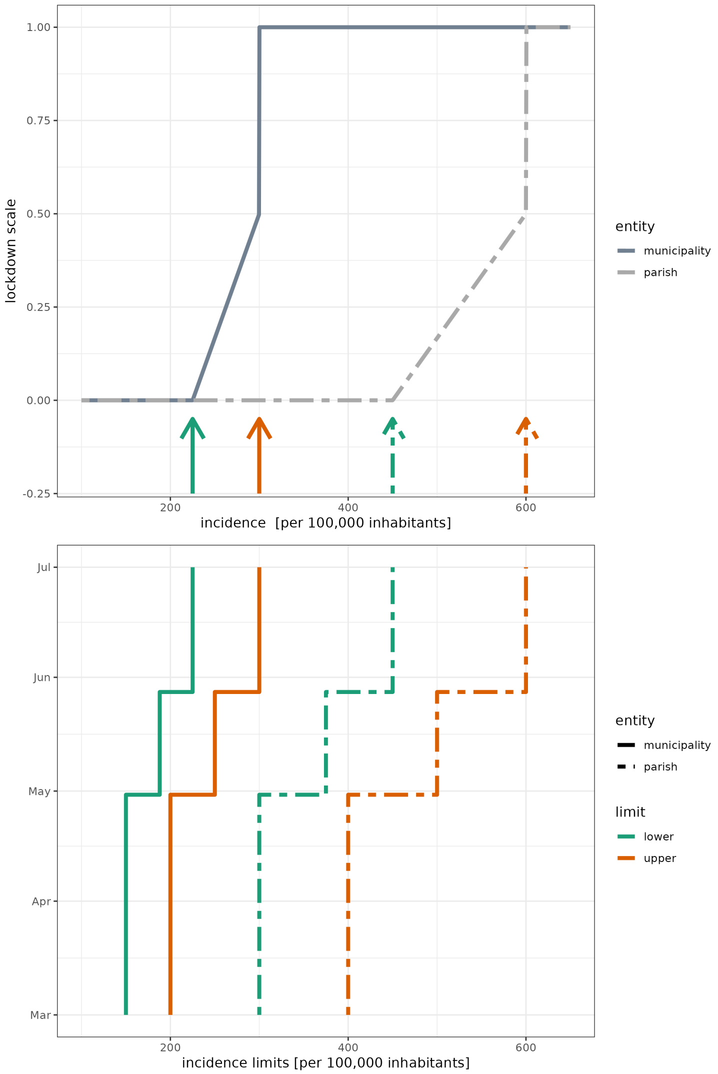

Supplementary Figure A6 Changing behavior close to lockdown limits (top), and changing limits of local lockdown over time (bottom). The upper incidence is the official mandated lockdown incidence, while the lower is the estimated limit as to when people will start changing behavior.

For the best possible isolation of the effect of temperature, the development of hospital admissions in Sweden's regions (län) during the period from April 6 to August 30, 2020 (weeks 15-35) is examined [18]. During this period, there were no major changes in the level of restrictions in Sweden, making it easier to separate the effect of temperature from changes in activity. Sweden also has relatively large temperature differences from north to south, further aiding in the separation of the temperature effect. The model also takes into

account population density in different regions, with the remaining variation assumed to be described solely by the temperature proxy. The findings show that there was a reduction in transmission risk of approximately 50% from April to August in Sweden without any changes in the level of restrictions [19]. The model demonstrates how the transmission rate correlates with temperatures, allowing for the application of Danish temperature data to the Swedish model. Since absolute values of the $\beta(T)$ parameter cannot be used due to different levels of restrictions in Denmark and Sweden, they are converted to relative beta values. Using the model for Sweden on Danish data, it is estimated that the reduction in transmission risk from mid-March to mid-April 2020 was approximately 30% if the values for the Danish climate normal are used [20]. For 2021 actual temperatures which results in a maximal reduction of 47% of the transmission rate towards the end of the study period.

It is subject to large uncertainty to directly apply the model from Sweden to the situation in Denmark. Denmark had a much higher testing frequency than Sweden and a pronounced use of face masks. These factors may mean that we have already achieved some of the effects that seasonal variation provided in Sweden. Furthermore, the Swedish data includes the Swedish school summer vacation, which is expected to affect behavior. For other respiratory illnesses, it has been shown that school holidays reduce the spread of infection [21]. This is supported by data from the HOPE project [22], which indicates that the number of contacts in Denmark was lower than in Sweden in the spring of 2020, while the level was comparable during the summer of 2020. This suggests that the seasonal effect will be smaller in Denmark than in Sweden in the spring and will converge more over the summer.

A figure showing the fit of the seasonal model to Sweden can be found in [23] page 22 (in Danish).

The functional form is based on a general logistic function in the form:

$$
\begin{aligned}
S(T) &= A + \frac{K - A}{\left(1 + \exp\left(-B(T - T_0)\right)\right)^{1/\nu}} \\
\beta(T) &= \frac{S(T)}{S(T_{\text{ref}})}
\end{aligned}
$$

where T references the maximum temperature on a given day, and T_{ref} the maximum temperature on a given reference day. The reference day used in this work is 1 January 2020. The exact values for the parameters used can be found in Supplementary Table A3, and $\beta(T)$ is depicted in Figure A7.

A.7 Sensitivity analysis

To investigate the effect of changing the infectious period, $1/\gamma_I$, the popIBM was recalibrated to run with ± 2 days the original value while transmission risk, β_0, was scaled so that the baseline would match observed data. Results are seen in Supplementary Figure A8 and Supplementary Table A4.

Supplementary Table A3 Parameters describing season function

Parameter	Value
A	0.31982266
K	0.05558284
B	0.10372262
T_0	11.4137904
ν	0.95672625

exact values reported

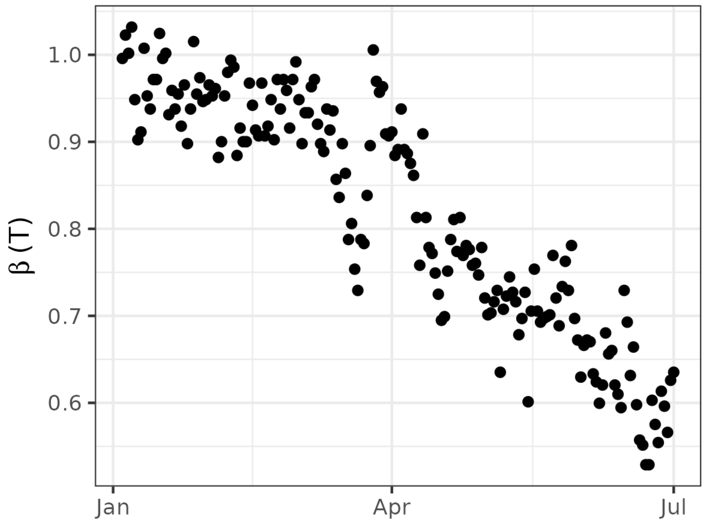

Supplementary Figure A7 The relative effect of the season as multiplied onto the transmission risk.

A.8 Additional Figures

Supplementary Table A4 Sensitivity analysis of changing $1/\gamma_I$

Scenario	$1/\gamma_I$ [days]	β_0 [1/days]	Lockdown [days]	Cumulative hosp. [1 / mil. inh.]	Change hosp. [%]
Data			Ref.	Ref.	Ref.
Mass tests	5.3	0.70	0.00 [-1.3; 1.6]	-65 [-210; 73]	-6.9 [-22; 7.7]
Limited tests	3.3	0.95	20 [19; 22]	1500 [1200; 1800]	160 [130; 190]
Limited tests	5.3	0.70	21 [20; 23]	1400 [1200; 1600]	150 [130; 170]
Limited tests	7.3	0.60	29 [27; 30]	1900 [1600; 2200]	200 [170; 230]

All these scenarios had local lockdowns. Numbers are medians, square brackets denotes range. inh. = inhabitants. mil. = million. hosp. = hospitalizations.

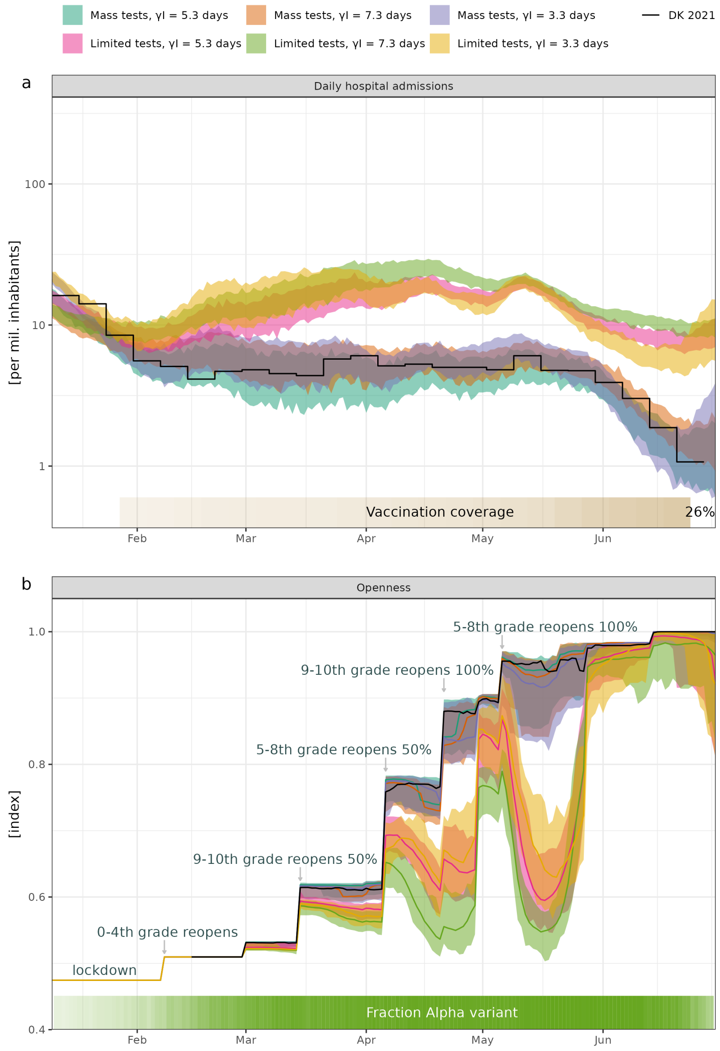

Supplementary Figure A8 Daily hospital admission and openness of society in the six simulated sensitivity scenarios. Labels in b figure only denote changes in school openings other restrictions may also have occurred. For the full list of changes in restrictions see [7]. Hospital admissions are averaged on a weekly basis to avoid weekend effects. The six scenarios, shown as colored bands, indicating the minimum and maximum of 100 simulation runs.

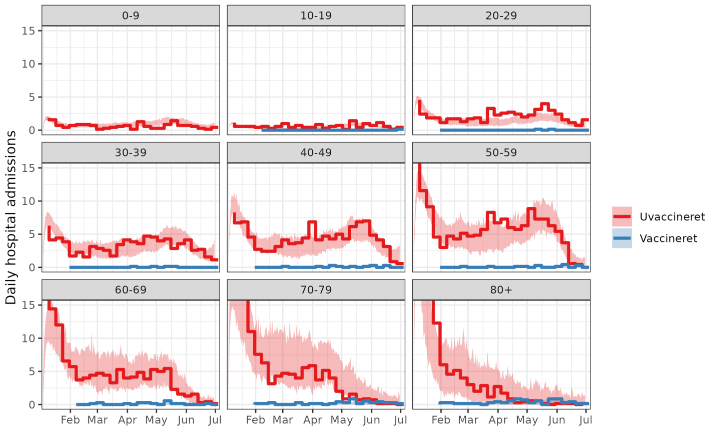

Supplementary Figure A9 Age and vaccination stratified daily hospital admissions for the baseline scenario compared to observed data. Solid lines are the observed admissions, and shaded areas are the simulation envelope.

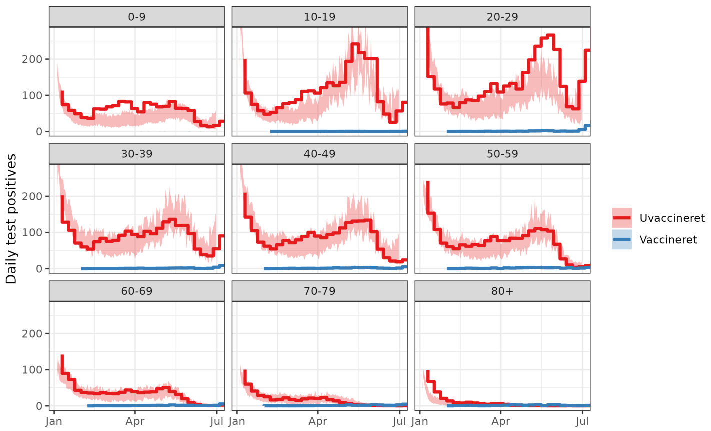

Supplementary Figure A10 Age and vaccination stratified daily test positives for the baseline scenario compared to observed data. The increase in test positives in the age group 20-29 following the end of the study period is likely due to the Delta variant and large gatherings following the success of the Danish National men's football team in the European Championship. Solid lines are the observed test positive rates, and shaded areas are the simulation envelope.

References

[1] Sundhedsstyrelsen: Vaccinationskalender. Accessed: 2023-06-07 (2021). https://www.sst.dk/da/udgivelser/2021/Vaccinationskalender

[2] Reilev, M., Olesen, M., Kildegaard, H., Støvring, H., Andersen, J.H., Hallas, J., Lund, L.C., Ladebo, L., Ernst, M.T., Damkier, P., *et al.*: Changing characteristics over time of individuals receiving covid-19 vaccines in denmark: A population-based descriptive study of vaccine uptake. Scandinavian Journal of Public Health **50**(6), 686–692 (2022)

[3] Wise, J.: Covid-19: European countries suspend use of Oxford-AstraZeneca vaccine after reports of blood clots. BMJ, 699 (2021). https://doi.org/10.1136/bmj.n699

[4] Klepac, P., Kucharski, A.J., Conlan, A.J., Kissler, S., Tang, M.L., Fry, H., Gog, J.R.: Contacts in context: large-scale setting-specific social mixing matrices from the bbc pandemic project. medRxiv (2020) https://www.medrxiv.org/content/early/2020/03/05/2020.02.16.20023754.full.pdf. https://doi.org/10.1101/2020.02.16.20023754

[5] Klepac, P., Kissler, S., Gog, J.: Contagion! the bbc four pandemic – the model behind the documentary. Epidemics **24**, 49–59 (2018). https://doi.org/10.1016/j.epidem.2018.03.003

[6] Mossong, J., Hens, N., Jit, M., Beutels, P., Auranen, K., Mikolajczyk, R., Massari, M., Salmaso, S., Tomba, G.S., Wallinga, J., Heijne, J., Sadkowska-Todys, M., Rosinska, M., Edmunds, W.J.: Social contacts and mixing patterns relevant to the spread of infectious diseases. PLoS medicine (2008). https://doi.org/10.1371/journal.pmed.0050074

[7] Munch, P.K., Espenhain, L., Hansen, C.H., Krause, T.G., Ethelberg, S.: Case-control study of activities associated with sars-cov-2 infection in an adult unvaccinated population and overview of societal covid-19 epidemic counter measures in denmark. PLOS ONE **17**(11), 1–16 (2022). https://doi.org/10.1371/journal.pone.0268849

[8] Statens Serum Institut: Municipality test positives. Accessed: 2023-06-30. https://files.ssi.dk/covid19/overvagning/data/overvaagningsdata-covid19-01072021-mw23

[9] Johansson, M.A., Quandelacy, T.M., Kada, S., Prasad, P.V., Steele, M., Brooks, J.T., Slayton, R.B., Biggerstaff, M., Butler, J.C.: SARS-CoV-2 Transmission From People Without COVID-19 Symptoms. JAMA Network Open **4**(1), 2035057–2035057 (2021) https://jamanetwork.com/journals/jamanetworkopen/articlepdf/2774707/johansson. https://doi.org/10.1001/jamanetworkopen.2020.35057

[10] Galmiche, S., Cortier, T., Charmet, T., Schaeffer, L., Chény, O., von Platen, C., Lévy, A., Martin, S., Omar, F., David, C., et al.: Sars-cov-2 incubation period across variants of concern, individual factors, and circumstances of infection in france: a case series analysis from the comcor study. The Lancet Microbe (2023)

[11] Kissler, S.M., Fauver, J.R., Mack, C., Tai, C.G., Breban, M.I., Watkins, A.E., Samant, R.M., Anderson, D.J., Metti, J., Khullar, G., *et al.*: Viral dynamics of sars-cov-2 variants in vaccinated and unvaccinated persons. New England Journal of Medicine **385**(26), 2489–2491 (2021)

[12] Polack, F.P., Thomas, S.J., Kitchin, N., Absalon, J., Gurtman, A., Lockhart, S., Perez, J.L., Pérez Marc, G., Moreira, E.D., Zerbini, C., *et al.*: Safety and efficacy of the bnt162b2 mrna covid-19 vaccine. New England journal of medicine **383**(27), 2603–2615 (2020)

[13] Nasreen, S., Chung, H., He, S., Brown, K.A., Gubbay, J.B., Buchan, S.A., Fell, D.B., Austin, P.C., Schwartz, K.L., Sundaram, M.E., *et al.*: Effectiveness of covid-19 vaccines against symptomatic sars-cov-2 infection and severe outcomes with variants of concern in ontario. Nature microbiology **7**(3), 379–385 (2022)

[14] Emary, K.R., Golubchik, T., Aley, P.K., Ariani, C.V., Angus, B., Bibi, S., Blane, B., Bonsall, D., Cicconi, P., Charlton, S., *et al.*: Efficacy of chadox1 ncov-19 (azd1222) vaccine against sars-cov-2 variant of concern 202012/01 (b. 1.1. 7): an exploratory analysis of a randomised controlled trial. The Lancet **397**(10282), 1351–1362 (2021)

[15] Ekspertgruppen for matematisk modellering af covid19: Teknisk baggrundsrapport den 26. marts 2021: 5. den ugentlige vaccineudrulning og forventet effekt af vacciner. Technical report, SSI (2021). https://covid19.ssi.dk/-/media/arkiv/subsites/covid19/modelberegninger/teknisk-baggrundsrapport-26032021.pdf

[16] UK Health Security Agency: Investigation of sars-cov-2 variants: Technical briefings. Technical report, UK Government (2020-2021). https://www.gov.uk/government/publications/investigation-of-sars-cov-2-variants-technical-briefings

[17] The Ministry of the Interior and Health: Ny automatisk model for lokale nedlukninger. Accessed: 2023-11-17 (2021). https://sum.dk/nyheder/2021/marts/ny-automatisk-model-for-lokale-nedlukninger

[18] Socialstyrelsen: Statistik om slutenvård av patienter med covid-19. https://www.socialstyrelsen.se/globalassets/1-globalt/covid-19-statistik/statistik-om-slutenvard-av-patienter-med-covid-19/statistik-covid19-inskrivna.xlsx (2020)

[19] Ludvigsson, J.F.: The first eight months of sweden's covid-19 strategy and the key actions and actors that were involved. Acta Paediatrica **109**(12), 2459–2471 (2020)

[20] Danmarks Meteorologiske Institut: Klimanormaler Danmark. https://www.dmi.dk/vejrarkiv/normaler-danmark/ (2023)

[21] Bjørnstad, O.N., Finkenstädt, B.F., Grenfell, B.T.: Dynamics of measles epidemics: Estimating scaling of transmission rates using a time series sir model. Ecological Monographs **72**(2), 169–184 (2002)

[22] Petersen, M.B.: HOPE Project. Accessed: 2023-06-19 (2020). https://hope-project.dk/#/about

[23] Græsbøll, K., Johnsen, M.G.: Teknisk baggrundsrapport den 26. marts 2021: Sammenhæng mellem temperatur og transmissionsrate for covid-19. Technical report, SSI (2021). https://covid19.ssi.dk/-/media/arkiv/subsites/covid19/modelberegninger/teknisk-baggrundsrapport-26032021.pdf
