# Supplementary material for: Digital twin simulation modelling shows that mass testing and local lockdowns effectively controlled COVID-19 in Denmark
Source: Commun Med (Lond). 2024 Oct 4;4:192. doi: 10.1038/s43856-024-00621-9 (PMC11452704; doi:10.1038/s43856-024-00621-9)
Supplement: Supplementary file 3 — Description of Additional Supplementary Files [file 43856_2024_621_MOESM3_ESM.pdf]

Description of Additional Supplementary Files

File name: Supplementary Data 1

Description: The supplementary data consist of six csv files containing all data and all simulated results shown in Figure 1. All files labelled *Data.csv has data that is also officially available through SSI (See section on Data availability for details), with the exception of the OpennessData.csv file which is the openness for the period as defined in the manuscript. All files labelled *Sim.csv are results from the simulations done for this manuscript. All files have a 'Date' column which specify the date on the figure. All *Sim.csv files have simID column which identify each unique simulated run (min-max bands on the figure can be obtained by taking min-max on any given day). There are two hosp*.csv files that contains data and simulations on hospitalisation incidence; two Openness*.csv files that contains data and simulations of the openness index; one AlphaData.csv file that contains fractions of the Alpha variant; and one VacData.csv file that contains the Vaccination coverage."
